# Perceptions of the Post First-Lockdown Era in the Current Covid-19 Pandemic: Quantitative and Qualitative Survey of the French Population

**DOI:** 10.3389/fpsyg.2021.668961

**Published:** 2021-06-28

**Authors:** Damien Fouques, Dana Castro, Marion Mouret, Sabrina Julien-Sweerts, Lucia Romo

**Affiliations:** ^1^CLIPSYD Laboratory UR 4430, Department of Clinical Psychology, Paris Nanterre University, Nanterre, France; ^2^Independent Researcher in Social Psychology, Paris, France; ^3^Department of Psychology, C2S EA 6291, Université de Reims Champagne Ardenne, Reims, France

**Keywords:** COVID-19 pandemic, lockdown context, post lockdown perception, anxiety, hope

## Abstract

**Introduction:** A pandemic with the severity of COVID-19 affects people's lives physically, as well as their daily routines, views of the world, and emotional balance. Lockdown is often an unpleasant experience due to a separation from loved ones, loss of freedom, and uncertainty over the disease status. To adjust, individuals and groups have had to adapt their perceptions of the event to the current scenario. This study aims to describe the perceptions of confined people on the changes occurring in their lives in the aftermath of the COVID-19 lockdown.

**Methods:** A total of 1,534 individuals (26.6% men; 73.4% women; mean age 41.6) responded to the questionnaire comprising 19 closed and five open-ended questions about the changes they anticipated in their lives in the immediate post-confinement era.

**Results:** Two definite groups appeared in the results: those who lived the confinement pleasantly, and those for which it was painful. They differ according to their confinement conditions and perceived degree of exposure to the virus. There seems to be a link for those who had a pleasant experience to a lower perceived exposure to the virus and less burdensome confinement conditions (young children, surface area, etc.). Lockdown conditions seem to influence the respondents' perceptions: a pleasant experience is associated with a vision of the society's evolution at large, and the care about its economic and professional progress; a painful one is associated more with focusing on the immediate needs of social support and personal well-being.

**Discussion:** Emotional experience during lockdown impacts the perception of its aftermath, with hope and anxiety becoming two ways of coping with uncertainty.

## Introduction

A viral pandemic, such as COVID-19, is unprecedented, especially for younger generations who have never experienced epidemics, famine, or war. Its rapid spread and high mortality rate affected people's lives physically, their daily routines, worldviews, and emotional balance, and globally, is expected to pose a mental health threat of great magnitude (Elhai et al., 2020). The health system gradually collapsed in France due to the tremendous increase in the number of COVID-19 cases, where hospital infrastructures were not able to manage or transfer patients with acute respiratory conditions. The contagiousness of COVID-19 necessitated a drastic social management to halt transmission, and an efficient way of protecting people was their isolation via a lockdown (Atalan, 2020). In France, with the aim of “flattening the curve,” the government isolation policies included bans on public gatherings, closures of academic institutions and public places, drastic national and international mobility restrictions, confinement at home with a limitation to leaving the house for a maximum of 1 h a day, and within a radius of 1 km from home. These measures resulted in situations such as “home schooling” for all children and adolescents, the massive introduction of teleworking, when possible, and the implementation of partial unemployment (with a financial compensation close to full-time salary) in situations unsuited for teleworking.

The lockdown situation in France started on March 17 and ended on May 11, 2020, lasting 8 weeks (57 days). An extended lockdown, such as this, is known to be anxiety-inducing because of fear of the contamination for oneself and others; high levels of uncertainty regarding the future; anger; family tensions; a limited sense of autonomy, occupational difficulties, boredom, and the loss of usual daily markers (Hartley et al., 2020). Literature on the psychological effects of the lockdown is divided into two main areas. The first addresses *the clinical perspective*, referring to its impact on mental and physical health. The second focuses on *the social perspective*, relating to people's beliefs, attitudes, and expectations toward political leaders, lockdown management, social distancing, and the lifting of restrictions.

The majority of the research conducted globally has been dedicated to the adverse psychological effects of the lockdown around the world (Lunn et al., 2020; The Lancet Infectious Diseases, 2020). Lockdown measures have led to negative changes in sleep quality, mental health problems including depression, intense symptoms of anxiety, and stress in vulnerable individuals (Brooks et al., 2020), increased suicidal thoughts or behaviors due to social distancing, feelings of disconnection, perception of social pain (Conejero et al., 2020), and fear of contamination by the virus and risk of death (Ornell et al., 2020).

Through a social lens, several authors aimed to understand the psychological effects of the lockdown on social connectedness, resilience, vulnerability in the face of adversity, and fear of social consequences (unemployment, bankruptcy …). Some demonstrate that people expect strong and cohesive responses to the pandemic from their national leaders, and that under these conditions they are likely to trust their politicians, scientists, police, and communities, and have a positive attitude toward them. Social belonging and support are critical to people's ability to cope and remain resilient in the face of a collective threat and fear, such as the impending recession leading to increased unemployment, changes to the future of many sectors of the economy, and other uncertainties (Sibley et al., 2020). Some other studies are researching the compliance with government policies and public perceptions of the effect of the containment with the aim at understanding people's worries about the pandemic and the heterogeneities in people's approval of these policies within individual countries (Sabat et al., 2020).

Authors were also concerned with the quality of perceived social support through the concept of loneliness. Loneliness functions as an alarm to motivate individuals to reconnect with others and show resilience in response to lockdown measures (Luchetti et al., 2020). Webster (2020) emphasizes the importance of provisioning relevant, sufficient, reassuring, and meaningful information to promote positive attitudes toward health and societal support.

The results stemming from clinical and psychosocial research pinpoint possible negative effects in the post lockdown era. Free festive activities are anticipated as a reaction to experienced loneliness. Anxious hypervigilance—sometimes linked with conspiracy beliefs (Georgiou et al., 2020)—remind people that the virus has not been eradicated, pointing to the still present fear of contamination and disease. The economic figures presented by most governments are likely to elicit worries about social hardships and financial crisis.

In addition, the French media (written press, television and radio news, social networks) relayed information from reports, interviews and expert opinions, which were therefore not empirical, delivering messages such as: “people who are alone or have a small living space, without easy access to the outside, will experience the lockdown more painfully than others....” These assertions should at least be tested.

Thus, what happens on a clinical and social level during the lockdown may have implications for the post-lockdown era. The lockdown and post-lockdown periods appear to be intimately linked, as the perception of the former can shape the perception of the latter. In uncertain times, people tend to adapt by imagining the future according to their current personal beliefs and theories. Therefore, combining the clinical and psychosocial perspectives is essential to identify the overall difficulties encountered by a country's population and to offer adjusted suggestions for action.

The aim of this study, which combines a psychosocial and a clinical approach, is to understand how people's experience of confinement is lived and how it can influence their perception of the future. In this way, we hope to help promote better well-being in the event of future lockdown. It will explore individuals' perceptions of the lockdown according to their personal situations and collect their perceptions of the post-lockdown era by examining, during the containment period, their anticipatory thoughts and beliefs about the changes that will affect their lives in the aftermath of the lockdown.

## Materials and Methods

### Procedure

To meet the study's objectives, we implemented a two-sided approach. A questionnaire-based part to investigate the real conditions and background of the lockdown (quantitative data). We generated items and chose to assess some of them using numerical rating scales (NRS) as we could not find validated instruments to operationalize our variables (see below for more details).

Then, five open-ended questions were proposed to obtain qualitative data about the perceptions of the post lockdown era from individual, social, sanitary, and economic points of view, according to research methodology on social representation. The obtained data were examined through a quantitative statistical analysis.

### The Participants

The participants were all French, over 18, and had all provided informed consent.

### Materials

See questionnaire on Supplementary Material. A two-part questionnaire was created and administered via the Qualtrics© secure platform to guarantee security and anonymity. The link to the study was shared via email (about 1,000 personal and professional contacts) and Facebook and LinkedIn social networks (about 1,500 contacts), a French psychological association and a national online magazine, health and generalist newspapers, using the snowball method. The 19-item questionnaire's first part, comprising 16 items, collected data about the respondent's main socio-demographic characteristics, background and lockdown conditions, dwelling surface, occupational activity (part or full-time work), and interpersonal context (living alone or with others). Three NRS (from 0 to 10) were added to measure respondents' feelings toward the lockdown (pleasant, painful) and the subjective perceived degree of exposition (perceived vulnerability) to COVID 19.

The questionnaire's second part was based on five open-ended questions, namely: “What do you think will happen in the post-lockdown period from a sanitary, social, economic, professional, personal, and interpersonal point of view?”

The questionnaire was created from four distinct sources of information: (1) available literature; (2) team members' personal experience; (3) experience of team members' social networks experience (colleagues, friends and relatives); (4) national media (TV news broadcasts, articles in national dailies…). The wording of the questions was drafted after an inductive analysis based on the notes and observations taken by all members of the research team from these sources. The finalization of questions was completed after three debriefing sessions in which a consensus on the wording and content was reached by all members of the research team. The total time for the questionnaire completion was <15 min.

### Data Analysis

The quantitative data were computed through descriptive statistics. The differences between subgroups and variables were tested by *t*-tests. Statistical analysis was performed with Jamovi® software.

The quantitative analysis of qualitative data was performed by importing verbalisations from the open-ended question, into the Iramuteq® software, used specifically for the treatment of qualitative data. This software uses the lexicometric analysis (Reinert, 2008) to examine the frequency of use of the corpus' current words and to envision the discursive material by having access to the representational content of the words. This software offers a textual content analysis using descending hierarchical classification that aims at splitting up groups of words, allowing an understanding of the hierarchical relationship between them, and observing the relationships between words within segments of the text defined by the software (Avelar et al., 2019).

## Results

### Main Characteristics of the Sample

As shown in Table 1, there were 1534 respondent subjects (73.4% women, 26.6% men), highly educated, aged from 18 to 96 with a mean age of 41.6. 31.5% were below 30, and 14% are above 60.

**Table 1:** Sample's socio-demographic characteristics and lockdown context.

**Variables**	**Percentage—Size (*N*)**
**Sample size** **=** **1,534**	
**Gender**	
Women	73.3% (1,125)
*Pregnant*	2.4% (27)
Men	26.6% (408)
Other	0.1% (1)
**Age**	
Years old, mean (*SD*) range	41.6 (15.3) 18–96
Below 30	31.47% (446)
From 31 to 59	54.55% (773)
Above 60	13.97% (198)
**Education level**	
Years, mean (*SD*) range	16.2 (2.4) 7–20
9 or less	1.05% (16)
10–12	9.91% (151)
13–15	22.78% (347)
Above 16	66.25% (1,009)
**Occupation status before lockdown:**	
Working	69.3% (1,060)
Job seeker	3.6% (56)
Student	14.8% (226)
Retired	10.6% (162)
Other (e.g., at-home parent)	1.7% (26)
**In activity**	
Before COVID	78.7% (1,207)
Still working	80.1% at the same rhythm
Stopped working	19.9%
Risk of exposure to COVID due to profession	14.7% (217)
**Declared virus experience**	
Declare infected	5.6% (65)
Declare not infected	22.3% (261)
Don't know	27.1% (357)
Relative or acquaintance infected	45.0% (526)
**Confinement social context**	
Alone	15.4% (235)
Couple	28.5% (435)
With a child under 18	23.3% (276)
With friends or sharing a flat	4.5% (68)
Number of people confined with (Mean, *DS*; mode)	1.96 (2.4); 1
**Dwelling surface (*****m**^**2**^**/p*****)**	
<10	0.8% (12)
11–20	14% (198)
>20	85.2% (1,213)
**Outdoor**	
No outdoor space	13.9% (213)
Outdoor space (balcony, private yard, garden)	86.1% (1,381)
**Outing's frequency**	
Once or more daily	32.9% (505)
2-3 times per week	33.6% (516)
Once or less per week	27.2 % (417)
Never	6.3% (96)

*Due to missing values, available-case analyses were performed*.

Eighty-four percent of the total sample were active (working or studying) prior the lockdown and 80% of them went on with their professional activity during the containment period.

### Respondents' Feeling During the Lockdown

#### Painful Experience of the Lockdown

50.6% (*N* = 644) found the lockdown either as or more painful than pleasant, compared to 49.4% (*N* = 629) who found it more pleasant than painful.

Overall, 16.8% of respondents experienced a painful lockdown (*N* = 240).

To better describe the factors associated with a negative lockdown experience, we split the sample into two sub-groups: the “painful group” who acknowledged the “painful lockdown experience” (NAS ≥ 7) vs. the “not painful” group (NAS <7) (see Table 2).

**Table 2:** Comparison between the 16.8% of subjects who experienced the lockdown as very painfully with the 83.2 % who did not.

	**Very painful (≥7)**		**Not so painful (<7)**				
	***N*** **=** **240**		***N*** **=** **1,189**				
	**Mean**	**(*SD*)**	**Mean**	**(*SD*)**	***t***	***p*-value**	**Cohen's *d***
Age	38.7	(14.2)	42.1	(15.6)	−3.041	0.0024	−0.22
Youngest child age	15.6	(12.3)	19.6	(12.7)	−2.97	0.0031	−0.32
Oldest child age	20.3	(12.6)	24.9	(13.5)	−3.152	0.0017	−0.34
Feeling of exposure	3.3	(2.6)	2.8	(2.5)	2.836	0.0046	0.20
Dwelling surface (*m^2^/p*)	39.17	(29.79)	44.25	(31.48)	−2.228	0.0261	−0.16

Compared with the other subjects, people who experienced the lockdown in a painful way were younger, parents of younger children, felt more exposed to the virus and lived in smaller surfaces.

#### Pleasant Experience of the Lockdown

29.2% of respondents experienced lockdown in a very pleasant way (*N* = 399).

None of the variables from Table 1 were able to explain any difference between the “not very pleasant” vs. the “very pleasant” groups (results not presented).

#### Very Pleasant vs. Very Painful Lockdown Experience

To go further, we decided to deepen the analysis by comparing the two contrasted groups: “very painful” and “very pleasant” (see Table 3).

**Table 3:** Comparison between contrasted groups “very painful lockdown (VPL)” vs. “very pleasant lockdown (VCL).”

	**VPL**		**VCL**				
	***N*** **=** **229**		***N*** **=** **399**				
**Variable**	**Mean**	**(*SD*)**	**Mean**	**(*SD*)**	***t***	***p*-value**	**Cohen's *d***
Youngest child age	15.5	(12.3)	19.6	(12.6)	−2.6	0.001	−0.33
Oldest child age	20.2	(12.6)	25.1	(13.5)	−2.9	0.004	−0.38
Perceived vulnerability of exposure	3.4	(2.6)	2.5	(2.5)	4.3	0.00002	0.35
Dwelling surface (*m^2^/p*)	39.0	(29.8)	44.6	(31.2)	−2.1	0.035	−0.18

*Ten respondents who had declared that lockdown was very comfortable and very painful were excluded from the analysis*.

Results show approximately the same differences as those observed between “painful” and “not painful.” The only variable that was not discriminatory here was “age.”

There is no statistical link between these results and the duration of the lockdown (pleasant × duration: Pearson's *r* = 0.02; *p* = 0.56; painful × duration: Pearson's *r* = −0.04 *p* = 0.13).

#### Summary of the Findings

Among our selected variables, the quantitative results suggest that perceptions of this lockdown as painful or pleasurable depend mainly on the age of the children, perceived vulnerability to COVID 19 and housing conditions. Respondents having had a comfortable experience of the lockdown are those who were confined with their teenagers or young adult children, in dwellings with a larger surface and reporting less vulnerability to Covid 19. Conversely, respondents with a painful experience of the lockdown are those who were confined with young children, in a smaller living surface and reporting more vulnerability to Covid 19.

### Post-Lockdown Respondents' Perception

**Figure 1:**
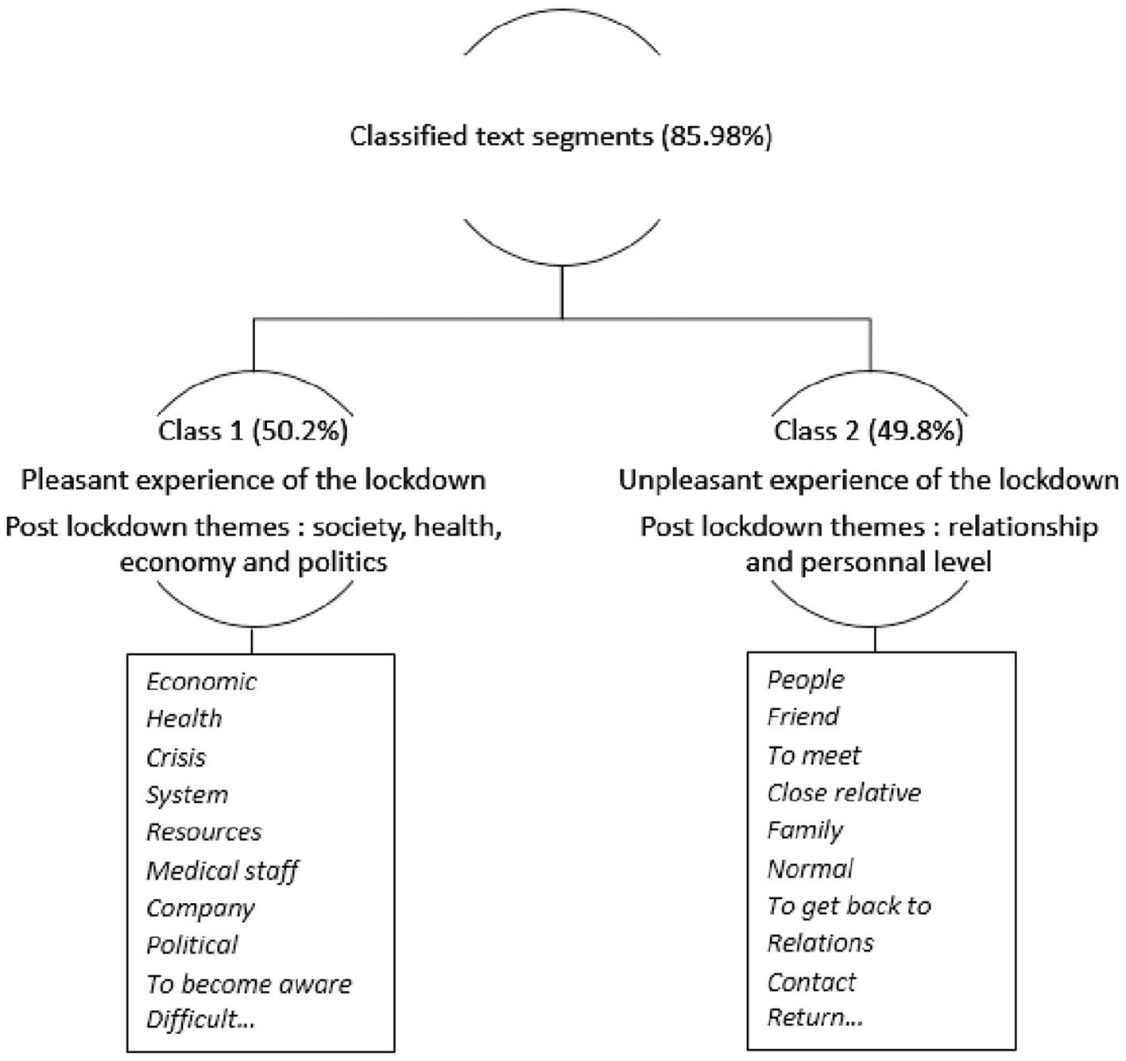
Dendrogram resulting from descending hierarchical classification corpus.

Given the results obtained from the quantitative data, we performed a textual analysis taking into account the variable “experience of the lockdown.” The aim of this analysis was to observe whether the participants discourse varied according to whether or not they had a pleasant experience of the lockdown. The textual analysis, resulting from the respondents written responses to the open-ended questions, showed two classes of words (85.86% of classified text segments emphasized by the Iramuteq® software). The first class (50.2%) was related to the epidemic's impact at societal, professional and sanitary levels, and the second (49.8%) to the social, personal, and relationship life. The words selected where those for which the χ^2^ of association to the two classes was over 15.20. There is no threshold for the χ^2^ choice. Tool words (prepositions, articles, etc.) and polysemous words were removed and only nouns, verbs, and adjectives were considered. Figure 1 shows the organization of the word classes.

#### Perceptions of the Post Lockdown by the Respondents of the Class 1

Class 1 is significantly associated with the responses of those who had experienced the lockdown as pleasant (χ^2^ = 7.08, *p* = 0.008) see Table 4. Class 1 lexical field is associated with foresights of post-lockdown society (χ^2^ = 166.6, *p* < 0.0001), health (χ^2^ = 127.28, *p* < 0.0001), and professional issues (χ^2^ = 23.92, *p* < 0.0001). Theses respondents were particularly loquacious about their idea of the post-confinement society changes at large, and about France's sanitary, economic, and professional adjustments. We describe each theme as follows.

**Table 4:** Description of the words associated with class 1 and the χ^2^ associated with the class.

**Associated**	**χ^**2**^ of association**	**Associated**	**χ^**2**^ of association**
**forms**	**(*p* <0.0001)**	**forms**	**(*p* <0.0001)**
Economic	252.36	Screening	15.89
Health	245.94	Wave	24.53
Crisis	184.79	Money	24.24
System	115.91	Employee	24.01
Resources	109.5	Salary	24.01
Medical staff	106.48	Tension	23.89
Company	106.07	Action	23.87
Political	88.79	Bankruptcy	23.87
To become aware	81.63	Patient	23.54
Difficult	79.38	National	23.01
To get	70.87	Investment	23.01
To hope for	67.6	Sanitary	22.93
Staff	66.16	Psychologist	22.92
Government	65.33	Increase	22.4
Financial	64.91	Care	22.16
Unemployment	63.55	Measure	22.03
Work	62.95	Work experience	21.87
Question	62.88	France	21.21
Public	61.95	Citizen	20.87
Professional	60.25	To expect	20.62
State	58.39	Psychological	20.49
Teleworking	58.24	Country	20.41
Increasing	57.86	Present	20.41
Difficulty	57.59	Working	20.04
Sector	57.24	Necessity	20.03
Demand	56.74	Necessary	19.66
Problem	51.02	Worry	19.66
Economy	50.97	Impact	19.31
Trade	49.0	Future	19.07
Society	48.05	Reform	18.88
Questioning	46.07	Recession	18.88
Job	41.38	Post	18.88
Risk	40.3	Way of life	18.87
Services	39.59	To be afraid of	18.86
Big	38.41	Field	18.11
Environmental	38.1	Liberal	17.88
Occupation	35.64	To allocate something to	17.88
Medical	35.16	Planet	17.72
Priority	34.6	Delay	17.16
Consequence	31.87	To boost	17.16
Budget	31.27	To get going again	16.93
Second	30.91	Worse	16.89
Material	29.98	Global	16.89
Numerous	28.33	Organization	16.54
Decision	27.98	Uncertainty	16.21
Management	27.98	Self-employed	16.08
Improvement	27.38	Lesson	16.08
Fall	26.99	Population	16.06
Rise	26.99	Inequality	15.9
Recognition	26.33	Epidemic	15.87
Mental	25.99	Precaution	15.86
Hospital	25.86	To work	15.68
Condition	25.78	Production	15.58
Major	25.0	State of emergency	15.26
Better	24.91	Local	15.23

##### Discourse on Society at Large

The respondents' perception of the changes occurring in the post lockdown era in the society at large, oscillate between the hope of positive societal shifts—in terms of ecology awareness and increased quality of life, and the fear that the situation will, at least, remain the same (in terms of social inequalities, individual or groups' aggressiveness or social tensions escalation), and at most, will become worse and will “furnish the government with excessive powers” leading to an authoritarian state.

##### Discourse on Sanitary Issues

Regarding this topic, respondents wavered between hope in collective changes toward health—the development of crisis plans or the renewal of health policies, and fear about the non-governmental response to the necessary evolution of the French health system; or the increase of traumatic psychological consequences such as panic disorders, anxiety, etc.

##### Discourse on Professional and Economic Issues

Regarding this topic, a percentage of respondents hope that the lockdown will act as a favorable time to reflect on work motivations and professional activities in the light of individual values and “life projects.” Others believe that the facilities of “teleworking and increased digitalization” will convey a better quality of a work-life balance.

They also fear that the economy will be impacted, mainly in the private sector, with “financial difficulties,” negative implications for young people's employability, unemployment, bankruptcies, layoffs, and more strain and pressure due to harder work conditions.

#### Perceptions of the Post Lockdown by the Respondents of the Class 2

Class 2 is significantly associated with the responses of those who had painfully experienced the lockdown (χ^2^ = 3.89, *p* = 0.049) see Table 5. Class 2 lexical field is associated with themes concerning the post-lockdown period at a relationship (χ^2^ = 106.78, *p* < 0.0001) and personal level (χ^2^ = 78.53, *p* < 0.0001). The comments of respondents who had a painful experience of lockdown are primarily associated with the use of social support in the post-lockdown period.

**Table 5:** Description of the words associated with class 2 and the χ^2^ associated with the class.

**Associated**	**χ^**2**^ of association**	**Associated**	**χ^**2**^ of association**
**forms**	**(*p* < 0.0001)**	**forms**	**(*p* < 0.0001)**
People	214.46	Distrustful	33.94
Friend	198.34	Hand	33.8
To meet	197.38	Relief	31.19
Close relative	164.42	Beginning	31.03
Family	145.96	Neighbor	30.56
Normal	125.28	To change	29.93
To get back to	104.91	To live	29.27
Relations	104.35	Careful	26.88
Contact	84.13	Child	26.55
Return	77.8	Reunion	25.41
To go out	71.94	Resumption	24.96
Pleasure	70.35	To infect	24.87
To take advantage of	64.44	To catch	23.87
Distrust	63.43	To be sick	23.75
Happy	63.26	To see	23.38
Joy	61.21	Rhythm	22.87
Habit	53.62	To refocus	22.27
Life	50.55	Solidarity	22.01
To be careful of	49.18	To become again	20.9
To feel like	48.55	To come closer	20.22
Bond	47.67	Colleague	19.97
Preventative measures	47.58	Encounter	19.23
To see again	44.51	Warm	19.23
Distance	44.3	Caution	19.12
Pleased	42.04	Exchange	18.89
Liberty	40.48	Usually	18.89
To be afraid of	40.33	Gathering	18.21
To appreciate	38.7	Hugging and kissing	18.21
Fear	37.6	To respect	17.56
To share	35.93	Human	16.09
Progressive	35.7	Parent	15.92
Celebration	34.49	Outing	15.51
Kiss	34.49	To be eager to	15.41
Mutual aid	34.07	Physical	15.26

##### Discourse on Relationships

While striving again to have close relationships, respondents oscillate between the hope that in the post lockdown, they will be able to meet their social desires: thirst of encounters, hugs, travels, etc.; and that the fear about the pandemic will continue distrust in people and fear of contagion, which will again impede the satisfaction of their needs.

##### Discourse on the Personal Level

At a personal level, respondents' perceptions alternated between the hope of living well again—freedom, physical contact, coming together with families and friends, and the perceived difficulties in the shift in adjusting to a new rhythm.

#### Summary of the Findings

The respondent's perceptions about changes occurring in their lives in the post-lockdown era are both positive and negative. The positives convey the belief in the beneficial evolution of the society, where the negatives point toward a future loaded with great difficulties in all life areas.

The respondents' lockdown experience models their post-lockdown perceptions: those who had a pleasant experienced are more prone to envision society's evolution at large and care about its economic and professional progress; those who had a painful experience are more focused on their immediate needs of social support and personal well-being.

## Discussion

The present study surveyed a sample of somewhat higher educated French citizens to capture their perceptions of the post-lockdown era at a personal and societal level, as well as its possible link to their lockdown conditions. The sample comprised mostly women, a finding common to other French studies on COVID-19 (see www.adaptation-institute.com). Most respondents did not feel at risk of contracting COVID-19, which is congruent with other international data (Bavel et al., 2020). Two definite groups came out from our two-sided study distinguished by the emotional experience toward the lockdown.

The first group experienced the lockdown as a pleasant event and had a low perceived vulnerability to COVID-19. Good confinement conditions appear to enhance life satisfaction in times of crisis, enabling people to develop better strategies for adapting positively to adversities such as the lockdown (Morales-Vives et al., 2020). Discharged from overwhelming daily hassles, the respondents of this group had a more intellectualized approach to society as a whole and the changes that might occur in the aftermath. Nevertheless, they expressed worries about the decrease of personal well-being because of the governmental economic and sanitary responses to the pandemic (Best et al., 2020; Sabat et al., 2020), social discontent (Kluth, 2020), economic recession, increased unemployment, and other uncertainties (McKibbin and Roshen, 2020).

The second group lived the lockdown as a painful experience, and had a high perceived vulnerability to COVID-19. Some of the respondents of this group were confined couples with small children, having exacerbated parenting responsibilities, who often must combine work, childcare, and home schooling (Best et al., 2020; Hartley et al., 2020). Others were people confined in lower quality living environments, and suffering from this situation (Glowacz and Schmits, 2020). Respondents in this second group had a more emotional approach to the post-lockdown era, which they see as a way of satisfying frustrated proximity needs through the renewal of close relationships and freedom to enjoy all aspects of life. The greater effect of the lockdown on respondents' daily life is associated with the need for social support and connectedness (Luchetti et al., 2020). Belton et al. (2020) shows that, if allowed, permitting interactions with people from other households is ranked as having the most positive impact, because social belonging and support is critical to people's ability to cope and remain resilient in extreme situations (Sibley et al., 2020).

Whether in an intellectualized or emotional way the two groups expressed simultaneously both hope and anxiety, which reflects the difficulty in planning for an unclear future. It highlights the impact of uncertainty, which is fundamentally a mental state, as a subjective, cognitive experience of humans rather than a feature of the objective, material world (Anderson et al., 2019). Hope is a multifaceted human attribute, and a natural coping reaction to extreme situations (Sibley et al., 2020). Typically categorized as a positive emotion, it often occurs in the midst of negative or uncertain circumstances (Bruininks, 2012). Hope is also a cognitive construct which translates the belief that a positive future outcome is possible (Luo et al., 2020). Hope promotes a greater sense of social connectedness (Sibley et al., 2020) and adherence to positive values such as benevolence and universality (Ma et al., 2019), and strengthens the link to groups that respondents belong to such as family, communities, or countries (Greenaway, 2020). Hope helps people cope with adverse situations, enables them to envision the future and therefore, decreases uncertainty

Anxiety and distrust with life in the post-lockdown era appears to be another reaction to the uncertainty about the future. Anxiety emerges as a response to imagined omens and dangers which are perceived to have only negative consequences (Lunn et al., 2020). In such situations, people often respond to threating events with suspicion, and tend to protect themselves by implementing avoidance behaviors. This anxiety creates the idea that life will be slow to return to normal (Belton et al., 2020) and supports awareness of social inequalities that are magnified by the suffering that the virus has “put on the shoulders of lower-status groups” (Iyer and Jetten, 2011). Hope and anxiety are the two facets of coping with uncertainty. They enable people to engage in action, even if the action is about avoidance, and gives them a sense of control over extreme situations.

The survey might have some useful practical implications. During the lockdown, and in its aftermath, there is evidence that some interventions, stemming from applied clinical and social psychology, successfully prevent or, at least, manage the negative effects of the lockdown (Lunn et al., 2020). We believe that the most important factor is preparing people for other possible confinement or crisis situations. Indeed, promoting psychological resilience could contribute to lowering emotional distress during pandemic and lockdown periods (Lenzo et al., 2020).

At a psychosocial level, although personal lockdown conditions can't be controlled, more flexibility might be recommended in the organization of telework to enable families to match and adapt their working hours around childcare situations. It is also important to enable people to better organize their lockdown conditions by allowing them more time to reach family homes, or share houses with friends or with romantic partners. Moreover, to help address feelings of uncertainty, coherent, precise and clear information must be displayed throughout all the government's announcements and in all domains, such as health, education, work, organizations, etc.

At a clinical level, psychological interventions support stress and anxiety management, emotional regulation, and relationship reorganization (Bavel et al., 2020), through psychotherapy, virtual support groups, therapeutic education webinars, virtual wellness breaks, dissemination of wellness guides, or implementation of virtual behavioral health webinars (Kaslow et al., 2020).

There are several strengths in the current study, including the assessment of post-lockdown perception and its design associating quantitative and qualitative data. There are also some limitations to note. First, as often happens with online surveys, the sample, despite its homogeneity, is a non-random one, and does not capture the perception of the entire French population. It over-represents a well-educated part of the population. Second, no information was gathered on the respondents' mental state or possible psychopathological status.

## Conclusion

The study has shown that post lockdown perceptions of a sample of confined French people are simultaneously colored with hope and anxiety, which partly depends on the social and practical conditions in which lockdown was passed. To help in these extreme conditions, social and behavioral sciences can provide valuable insights for managing the lockdown and post-lockdown periods (Bavel et al., 2020), as in both cases people “are the solution,” as people, through their individual and collective behavior, can reduce transmission of the virus and save lives (Jetten et al., 2020). To fulfill these goals, clinical and social psychologists must combine their efforts to transform the best evidence into practice and, simultaneously, place this practice and its associated experiences under the scrutiny of the best research.

## Data Availability Statement

The datasets presented in this article are not readily available because RGPD French law. Requests to access the datasets should be directed to dfouques@parisnanterre.fr.

## Ethics Statement

The studies involving human participants were reviewed and approved by Comité D'éthique de Recherche de l'UFR SPSE Université Paris Nanterre, France. Written informed consent for participation was not required for this study in accordance with the national legislation and the institutional requirements.

## Author Contributions

DF, DC, and LR designed the study and wrote the paper. SJ-S and DF conceived the survey. SJ-S collected data. DF, SJ-S, LR, and MM performed statistical analysis. DC and MM interpreted the data. All the authors collaborated in the writing of the final manuscript.

## Conflict of Interest

The authors declare that the research was conducted in the absence of any commercial or financial relationships that could be construed as a potential conflict of interest.
