# Supplementary material for: Perceptions of the Post First-Lockdown Era in the Current Covid-19 Pandemic: Quantitative and Qualitative Survey of the French Population
Source: Front Psychol. 2021 Jun 28;12:668961. doi: 10.3389/fpsyg.2021.668961 (PMC8273336; doi:10.3389/fpsyg.2021.668961)
Supplement: Supplementary file 1 [file Data_Sheet_1.docx]

Supplementary Material

**Post-lockdown perception survey questionnaire translated from French**

1. I am:

A man

A woman : Am I currently pregnant?

Yes

No

I do not know

Other

2. I am … years old

3. The number of years of study is calculated as follow: CP = 1; CM2 = 5; 5ème = 7; Seconde = 10; BAC = 12; Licence = 15; Master = 17; Doctorat = 20, etc.

I have the following number of years of study:

4. My marital status:

Married

Civil partnership

In a common-law relationship

Single

Widowed

Divorced

5. I have children:

No

Yes number of children :

please specify the age of the eldest:

please specify the age of the youngest:

6. My professional situation:

Student

Unemployed/looking for a job

Retired

In employment

Other (stay-at-home parent…)

7. Before lockdown, I had a professional activity:

No

Yes Full time

Part time : %

8.During the lockdown (currently), I’m working

No

Yes Full time

Part time : %

At home

No

Yes please indicate the percentage of time worked at home out of total working time : %

9. I have been working in a profession that exposes me to coronavirus since lockdown (carer, shopkeeper…):

Yes

No

10. I feel exposed to coronavirus. On scale from 0 (not at all exposed) to 10 (the most I can imagine) please, place the cursor on the corresponding number:

| 0 | 1 | 2 | 3 | 4 | 5 | 6 | 7 | 8 | 9 | 10 |
| --- | --- | --- | --- | --- | --- | --- | --- | --- | --- | --- |

11. I am or have been affected by coronavirus:

Yes, I am sur of it (test done) : I still have symtoms : Yes No

Yes, my doctor is almost certain : I still have symtoms : Yes No

No

I don’t know

Not personally but close people (family members, friends, colleagues)

Not personally, but acquaintances (friends of friends, neighbours in the building…)

12. What are my conditions of lockdown?

I am confined:

Alone

As a couple

With close family (spouse, children) - if Q6 “yes” : With all of my children

With some of them

Without my children

With extended family (uncle/grandparent…)

With friends

In a shared flat

Other

13. I am confined with … people without counting myself (e.g. 0 if I am alone):

14. Among the people I share my home with (several answer possible):

Some are or have affected by COVID 19

Some are still ill

Some have died

No one is affected

15. I am confined to an area of approximatively (square metres):

16. The place where I am confined has (several answer possible):

A balcony or terrace

A yard where I can go from time to time

A private garden

A communal garden where I can go from time to time

None of the above

17. I go out of my house:

Several times a day

Once a day

Once every 2 days

3 times a week

2 times a week

Once a week

Less than once a week

Not at all

If 0 = not at all and 10 = completely, to what extent do you agree with the following proposals? (Place the cursor under the number that best fit).

18. Lockdown is painful for me:

| 0 | 1 | 2 | 3 | 4 | 5 | 6 | 7 | 8 | 9 | 10 |
| --- | --- | --- | --- | --- | --- | --- | --- | --- | --- | --- |

19. Lockdown is pleasant for me:

| 0 | 1 | 2 | 3 | 4 | 5 | 6 | 7 | 8 | 9 | 10 |
| --- | --- | --- | --- | --- | --- | --- | --- | --- | --- | --- |

**Open-ended questions:**

What do you think the post-lockdown period will be like? Feel free to express things as you see them. All answers are good because it is your opinion that interests us!

20. What do you this the post-lockdown period will be like in terms of health?

21. What do you think the post-lockdown period will be like for society?

22. What do you think the post-lockdown period will be like on a personal level?

23. What do you think the post-lockdown period will be like at the professional level?

24. What do you think the post-lockdown period will be like in terms of human relations?
